# Lived illness experience in fibromyalgia

**DOI:** 10.1192/j.eurpsy.2026.10342

**Published:** 2026-07-31

**Authors:** C. M. Esposito

**Affiliations:** 1 Department of Neurosciences and Mental Health, Fondazione IRCCS Ospedale Maggiore Policlinico, Milan; 2 Department of Health Sciences, University of Florence, Florence, Italy

## Abstract

**Background:**

Fibromyalgia (FM) profoundly affects not only physical functioning but also the lived experience of time, space, the body, selfhood, and relationships. Although many qualitative studies describe these challenges, their heterogeneity makes it difficult to identify common experiential patterns.

**Methods:**

We conducted a qualitative meta-analysis of 69 studies reporting first-person accounts of adults diagnosed with FM. Following PRISMA-ScR guidelines, we extracted patient quotations and analyzed them using Consensual Qualitative Research (CQR), incorporating triangulation, consensus-building, reflexive dialogue, and external auditing.

**Results:**

Across studies, FM emerged as a disruption of core experiential structures. Patients described collapsed futurity, fluctuating daily rhythms, and a biographical split between “before” and “after” illness. Their lived space narrowed due to pain, immobility, and fear of unpredictability. Embodiment became marked by pervasive pain, exhaustion, and bodily estrangement. Selfhood was threatened by loss of roles and diminished agency, while invisibility, disbelief, and isolation profoundly shaped intersubjective experience. Diagnosis brought both relief and misrecognition, sometimes fostering over-identification with the label.

**Conclusion:**

FM constitutes an existential transformation rather than solely a somatic condition. Clinicians should adopt phenomenologically informed, person-centred approaches that recognize patients’ lived experiences, avoid reductive interpretations, and support processes of validation, bodily reconnection, and identity reconstruction.

**Disclosure of Interest:**

None Declared

